# Migration is the driving force of rapid aging in Puerto Rico: A Research Brief

**DOI:** 10.1007/s11113-021-09683-2

**Published:** 2021-10-30

**Authors:** Amílcar Matos-Moreno, Alexis R. Santos-Lozada, Neil Mehta, Carlos F. Mendes de Leon, Félice Lê-Scherban, Amélia A. De Lima Friche

**Affiliations:** 1. Department of Epidemiology and Center for Social Epidemiology and Population Health, School of Public Health, University of Michigan, Ann Arbor, MI; 2. Population Research Institute, Pennsylvania State University. University Park, PA; 3. Department of Human Development and Family Studies, Pennsylvania State University. University Park, PA; 4. Department of Preventive Medicine & Population Health, School of Medicine, University of Texas Medical Branch. Galveston, TX; 5. Department of Epidemiology and Biostatistics, Dornsife School of Public Health, Drexel University. Philadelphia, PA; 6. Drexel Urban Health Collaborative, Drexel University. Philadelphia, PA; 7. Observatory for Urban Health in Belo Horizonte, School of Medicine, Federal University of Minas Gerais. Minas Gerais, BR

**Keywords:** Puerto Rico, Population Composition, Rapid Aging, Migration, Demographic Transitions

## Abstract

The combined effects of declining fertility and increased longevity have accelerated population aging in different parts of the world. Unlike other countries, Puerto Rico is also experiencing unprecedented levels of working-age out-migration. The full impact of high out-migration on Puerto Rican demography is not fully understood. Placing Puerto Rico’s aging process in an international context is useful in identifying the role out-migration is having on the accelerated aging of the Puerto Rican society. Using the World Population Prospects 2019 estimates, we compared the pattern of rapid aging found for Puerto Rico with the trajectories of six other countries with the highest population of 65+ in the World, Europe, and the Caribbean from 1960 to 2020. Prior to 2010, the aging process in Puerto Rico was comparable to the other countries. After 2010, the percent of the population over 65 years in Puerto Rico nearly doubled from 11% to 21%. The nearly doubling of the percent of older adults is not observed in any of the comparison countries. We find that the rapid aging of Puerto Rico, changing from a linear trend to an exponential one, is a result of accelerating levels of out-migration, which is concentrated in the working-age population.

## Introduction

In 2010, Puerto Rico experienced its first instance of population decline in recorded history (Figueroa-Rodríguez et al. 2012; Mora et al. 2017). According to the decennial census count, the population reduced from 3,808,610 to 3,725,789 people between 2000 and 2010, which is equivalent to a 2.2% decline (U.S. Census Bureau 2000, 2010). This decline was the result of decreased birth rates to below replacement levels since 1997, and the out-migration of approximately 390,507 people between 2000 and 2010 (Departamento de Salud de Puerto Rico 1997; Santos-Lozada et al. 2020). The 2020 decennial count indicated the population of Puerto Rico was 3,285,874 people as of April 1, 2020; a decline of 439,915, which is equivalent to a 11.8% reduction (U.S. Census Bureau 2020). While migration has been recorded at significant levels in the past (i.e. 1950 Mass Migration) the pace of population growth continued, and the population between ages 20 to 64 contributed to this growth (Senior 1953; Caraballo-Cueto 2020). The 2000-2010 growth pattern diverges from past ones; it constitutes the first instance in the last 70 years where growth was concentrated in the population aged 65 and older, and where the working age population declined. This pattern has continued during the 2010-2020 decade (Keenan and Hauer 2020). Continued monitoring of the changes in population composition is essential to generating fiscal plans, informing ongoing recovery efforts from Hurricanes Irma and María, and estimating the need for government and public services. Furthermore, the changes in the population age structure, happening parallel to this decline, raise questions about the sustainability of government operations and about the expected demand for educational and healthcare services.

Recent literature on the demography of Puerto Rico has focused on mortality and migration following Hurricanes Irma and Maria in 2017 (DeWaard et al. 2020; Sandberg et al. 2019). These studies focused on estimating migration, including the influence of economic shocks, and the settlement of Puerto Ricans in the United States (Alexander et al. 2019; Santos-Lozada et al. 2020). Despite the well-established interest in the study of population size, mortality, and migration, little attention has been paid to changes in the age-composition of Puerto Rico. A 2018 report by the Puerto Rico Institute of Statistics found that 93% of all out-migrants in the prior 11 years were under the age of 65 (Velazquez-Estrada 2018). Moreover, it estimated the median age of out-migrants to be 30 years old; this has remained relatively stable since 2007. The age patterning of out-migration has resulted in a reduction of the number of persons under 65 years in Puerto Rico. This reduction results in the population over 65 representing a higher proportion of the population, shaping the rapid aging of Puerto Rico

In this research brief, we provide the first overview of rapid aging in Puerto Rico by documenting changes in the percent of the population 65 years and older from 1960 to 2020. To highlight the distinct pace of aging in Puerto Rico, we compare these changes and patterns in fertility and mortality with six countries: Trinidad and Tobago, Spain, Italy, Cuba, Japan, and Jamaica. We also describe trajectories of net migration rates of these countries for the same period to better understand the relationship between migration and aging. Finally, we discuss the broader effects of out-migration and rapid aging on Puerto Rican population composition.

## Data and Methods

We accessed the following indicators from the 2019 World Population Prospects (WPP): total population by 5-year age groups, Crude Birth Rate (CBR), Crude Death Rate (CDR), and Net Migration Rate (NMR) between 1960 and 2020 (United Nations 2019). The CBR, CDR, and NMR are presented per 1,000 people. The WPP, prepared and published by the Population Division of the Department of Economic and Social Affairs of the United Nations, compiles and tracks annual demographic characteristics for nations throughout the world. To assess whether the progression in population aging in Puerto Rico followed standard patterns, we selected two countries with the highest percent of people over 65 years old in 2020 (Japan and Italy), the country with the highest percent of people over 65 years old in Europe following Italy (Spain), the two countries with the highest percent of people over 65 years old in the Caribbean (Jamaica and Cuba), and the Caribbean country with the highest negative NMR following Puerto Rico and Jamaica (Trinidad and Tobago). Demographic indicators for Puerto Rico and the comparison countries in 2020 are presented in Table 1.

First, we compared the trends in the percent of the population over 65 years. Second, we studied the patterns in demographic processes that have shaped population composition: CBR, CDR, and NMR. Third, we studied differences in age structure between 2010 and 2020 to illustrate the effects of out-migration.

## Results

**Figure 1.**
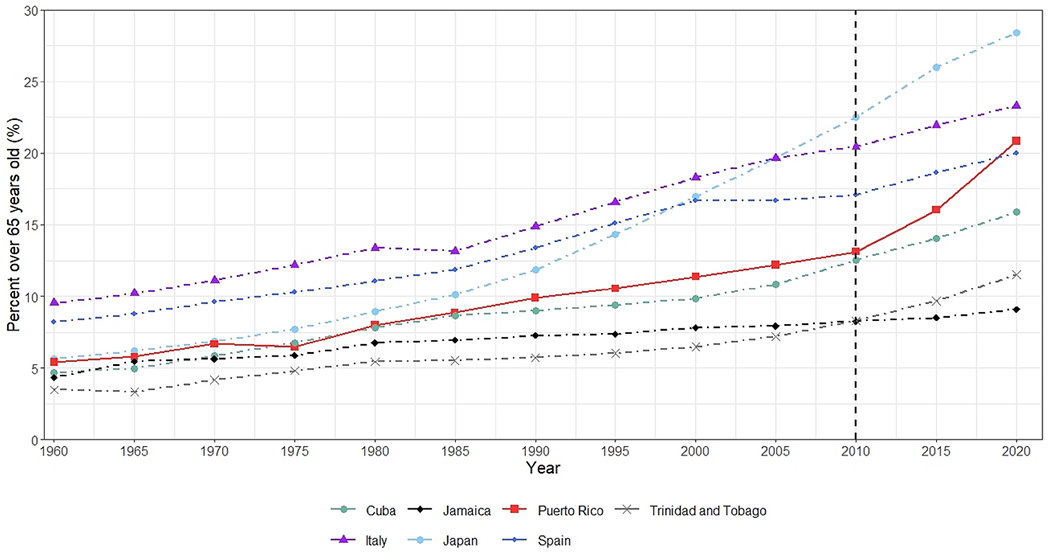
Percent of the population aged 65 years and older for Puerto Rico and selected comparison countries 1960-2020. Data from the United Nations World Population Prospects 2019.

Figure 1 shows the percent of the population over age 65 from 1960 to 2020 putting into context the exceptional pace of aging in Puerto Rico after 2010. Prior to 2010, the growth of Puerto Rico’s aged 65+ population was similar to that of Cuba, Italy, and Spain. Puerto Rico experienced a sudden increase in the percent 65+ from 13.1% in 2010 to 20.8% in 2020. By 2020, Puerto Rico had a higher percent of the population 65+ than Spain. In 2000, the percentage aged 65+ was 11.4%. Thus, Puerto Rico’s population aged 65+ doubled in only 20 years. In contrast, it took 30 years for the population aged 65+ of Spain to double and 35 years for Italy. The sudden increase in the population aged 65+ did not occur for Cuba, Trinidad and Tobago, or for Jamaica; their rate of aging has accelerated over time but at a much slower pace compared to Puerto Rico. Furthermore, Japan experienced an acceleration in pace around 1990, this increase exacerbated the gap in the aging pace when compared to Puerto Rico. Puerto Rico experienced an acceleration in the aging pace in 2010 and doubled its percent of the population aged 65+ in 10 years, whereas the doubling for Japan took approximately 23 years (1997- 2020). If Puerto Rico’s aging pace continues, the percent of the population aged 65+ could reach levels comparable to Japan in the future.

**Figure 2.**
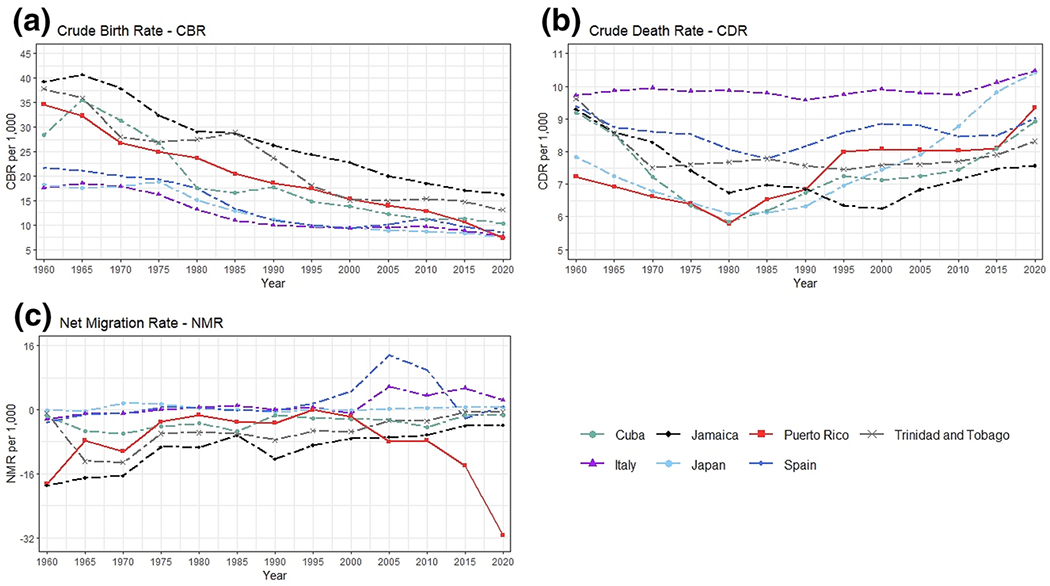
Evolution of demographic processes for Puerto Rico (solid-red) and comparison countries (dashed) 1960-2020. Panels show the Crude Birth Rate (CBR), Crude Death Rate (CDR) and Net Migration Rate (NMR). Data come from the United Nations World Population Prospects 2019.

Figure 2 shows trends in CBR, CDR, and NMR. Puerto Rico and the comparison countries experienced a decrease in the CBR between 1960 and 2020 (Panel a), a decrease that is consistent with developed countries and the demographic transition process. Puerto Rico’s declining CBR has been at a similar pace as that of Jamaica. Trinidad and Tobago also experienced a decline; however, it attenuated after 2000. In Panel b, we present the progression of CDR. Puerto Rico and the comparison countries have experienced an increase in CDR. Puerto Rico’s CDR increase attenuated between 1995 and 2015 in contrast to other countries.

Because we find consistent patterns in CBR and CDR, we expect differences in migration to be the primary driver of variations in changes in the population composition (Panel c). From 2000 to the present, Puerto Rico experienced substantial out-migration resulting in a higher negative NMR. In 2005, Puerto Rico lost approximately eight persons per 1,000 residents (NMR= −8). By 2020, it is estimated that Puerto Rico is losing 31 persons per 1,000 residents (NMR= −31). In contrast, after 2000, most comparison countries experienced small changes in NMR, either positive or close to zero. Italy and Spain experienced an increase in the NMR during the 2000’s decade due to being the preferred destinations for immigrants in Europe (Uribe-Exteberria et al. 2013).

The estimates of the demographic indicators for Trinidad and Tobago, Cuba, and Jamaica are the most comparable to Puerto Rico. The three countries exhibited similar NMR before 2000, a constant decrease in CBR, and a slight but constant increase in the percent of older adults. Given the proximity of these trajectories, we would expect these countries to exhibit consistent patterns in population aging. Nevertheless, the difference in population aging between Puerto Rico and the comparison countries is noticeable after 2010. Puerto Rico suddenly experienced an accelerated increase in the percent of older adults. This phenomenon is primarily driven by the increase in the negative NMR in Puerto Rico, which was not experienced by Trinidad and Tobago, Cuba, nor Jamaica.

**Figure 3.**
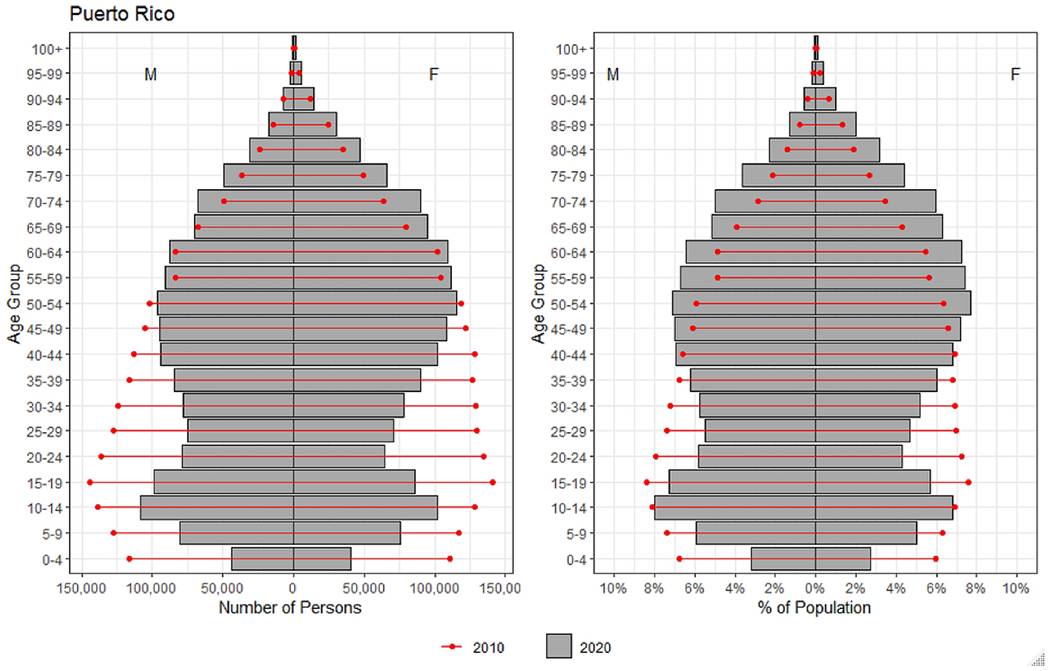
Population Pyramids for Puerto Rico in 2010 (red) and 2020 (gray). Showing the number of persons (left panel) and percent of the population (right panel) by age and sex. Data come from the United Nations World Population Prospects 2019.

**Figure 4.**
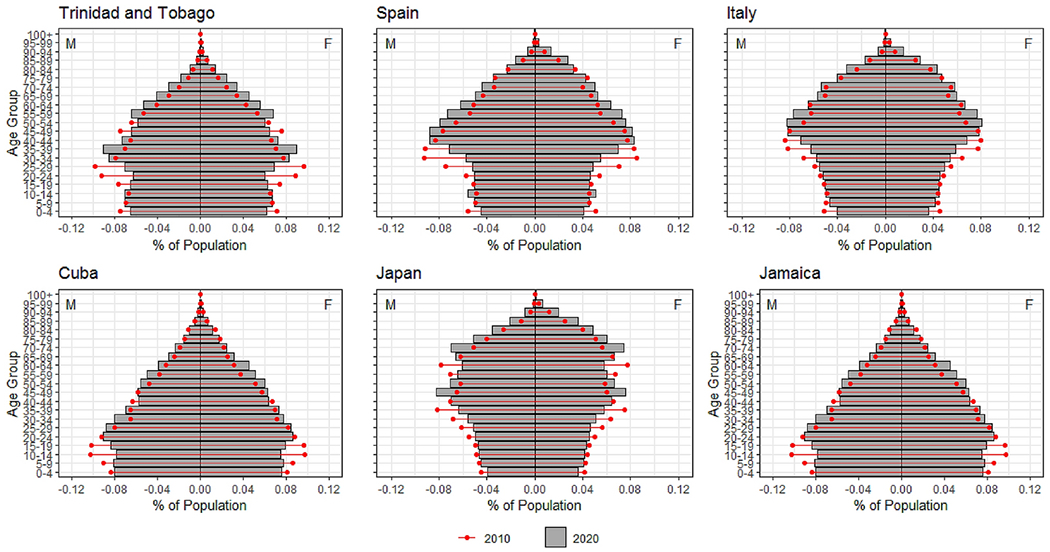
Population Pyramids for 2010 (red) and 2020 (gray) for comparison countries. Showing the percent of the population by age and sex. Data come from the United Nations World Population Prospects 2019.

In Figure 3 we show the population pyramid for Puerto Rico in 2010 and 2020 to illustrate changes in the age and sex composition. In figure 4 we present the corresponding population pyramids for the comparison countries. Puerto Rico, like other countries, is *aging from the base* as the result of the decline in CBR. Every country included in our analysis experienced this pattern. In the case of Puerto Rico, however, we observe that by 2020 the working-age population considerably declines in comparison to 2010. We term this pattern *aging through compression*, where migration reduces the working-age population relative to adjacent age groups and/or to the corresponding age-group in the previous period. Aging through compression is also evident in Trinidad and Tobago, but to a lesser degree compared to Puerto Rico. The increase in the percent of the population aged 65+, known as *aging from the top*, is observed for Puerto Rico and the comparison countries. Nevertheless, the comparison countries did not experience an expansion as noticeable as the one observed for Puerto Rico over the last 10 years. While every country experienced aging from the top and the base, in both instances Puerto Rico exceeds the other countries. Further, we only observe aging through compression in Puerto Rico.

## Conclusion

In this research brief, we provide a description of rapid aging in Puerto Rico within an international context. We compared Puerto Rico’s demographic processes to countries with high levels of population aging in the World, Europe, and the Caribbean. Puerto Rico and all countries within this study experienced an increase in the percent of the population over age 65 from 1960 to 2020. They also have similar trajectories of CBR and CDR, although the levels of these indicators differed. The population over age 65 years in Puerto Rico grew at a much faster pace after 2010 in parallel to the rapid increase in out-migration of the working-age population.

Previous studies have demonstrated that Puerto Rico’s socioeconomic characteristics are more similar to low-income than high-income countries, but its demographic characteristics resemble those of high-income countries (Mora et al. 2017). However, many of the economic indicators for Puerto Rico like the poverty rate, and unemployment rate shows Puerto Rico at a disadvantage relative to high-income countries. Puerto Rico’s situation is unique in the sense that it has unrestricted out-migration to the U.S. Given the political status of the island with the United States (known as the Commonwealth of Puerto Rico), Puerto Ricans are U.S. born citizens who can travel to the U.S. without the need of Visas nor Passports. This unique situation coupled with the effects of economic instability and environmental disasters exacerbates population movement and underscores the importance of examining the contemporary patterns of migration and the effect of these demographic shocks on society more broadly (Livi-Bacci 2001). While beyond the scope of our research brief, future studies should approach this comparison through a regional perspective focusing on the experience of other Caribbean countries or territories.

The speed at which Puerto Rico has aged poses an enormous burden on the social, education, and healthcare sectors of an already economically exhausted island. The unbalanced migration is affecting social conditions like housing, healthcare services, and public transportation, among others; elements that affect directly and indirectly population health. Given that migration pertains predominantly to the working-age population, the economy undergoes a direct loss due to the reduction of human capital (Chen et al. 2018). This causes an increased economic burden for those remaining in Puerto Rico and has the potential of reducing the availability of public funds crucial to sustain essential health and social services needed by older adults. Previous studies have found an association between the reduction of health and social services and the deterioration of the health of older adults (Benmarhnia et al. 2014). Moreover, the impact is even greater among older adults with a disability, a population that represents half of older adults in Puerto Rico (Matos-Moreno and Mendes de Leon 2018).

What does the future hold for the population composition of Puerto Rico? If the out-migration of younger age groups continues unabated, the proportion of population 65+ will keep growing at an accelerated pace, probably surpassing other countries that have achieved higher life expectancies and lower CBRs. We argue that the consequences of these demographic transformations may be understated in recent governmental policies as the changes in population composition have accelerated in recent times and are projected to continue (Keenan and Hauer 2020). It will be essential to observe whether migration patterns persist in the coming years, and to urge the Puerto Rican and U.S. governments to address it and plan for the needs of its aging population.

## Figures and Tables

**Table 1.** Demographic indicators for Puerto Rico and comparison countries in 2020.

Country	Population over 65 years (%)	Crude Birth Rate (CBR)	Crude Death Rate (CDR)	Net Migration Rate (NMR)	Region
Puerto Rico	20.83	7.4	9.3	−31.40	Caribbean
Japan	28.40	7.5	10.4	0.6	Asia
Cuba	15.90	10.2	8.9	−1.3	Caribbean
Jamaica	9.10	16.2	7.6	−3.9	Caribbean
Trinidad and Tobago	11.50	13.1	8.3	−0.6	Caribbean
Spain	19.97	8.5	9.0	0.9	Europe
Italy	23.30	7.6	10.5	2.5	Europe

**Source:** United Nations World Population Prospects, 2019

## Data Availability

The 2019 World Population Prospects estimates are available through U.N. Population Division (https://population.un.org/wpp/).
